# Boot camp approach to surgical residency preparation: feedback from a French university hospital

**DOI:** 10.1186/s12909-022-03745-5

**Published:** 2022-09-15

**Authors:** Etienne Buscail, Fabrice Muscari, Aurélien Hostalrich, Manon Bolzinger, Sandra Malavaud, Vincent Minville, Charlotte Martin, Magali Delhoste, Charles Henri Houze-Cerfon, Simon Buscail, Bruno Bastiani, Mathieu Roumiguié, Ariane Weyl, Nicolas Carrère, Olivier Abbo

**Affiliations:** 1 Department of Digestive Surgery, Toulouse University Hospital, Toulouse, France; 2 INSERM, U1220, University of Toulouse, Digestive Health Research Institute (IRSD), Toulouse, France; 3 Department of Digestive Surgery, Colo-Rectal Surgery Unit, CHU Rangueil, 1 avenue Jean Poulhès, TSA 50032, 31059 Toulouse Cedex 9, France; 4 Department of Vascular Surgery, Toulouse University Hospital, Toulouse, France; 5 Department of Pediatric Surgery, Toulouse University Hospital, Toulouse, France; 6 Department of Infection Control, Toulouse University Hospital, Toulouse, France; 7 Department of Anesthesiology and Intensive Care, Toulouse University Hospital, Toulouse, France; 8 Emergency Medical Service, Toulouse University Hospital, Toulouse, France; 9 The Toulouse Bar Association, Toulouse, France; 10 Toulouse Institute for Health Stimulation, Toulouse, France; 11 Department of Urology, Toulouse University Hospital, Toulouse, France; 12 Department of Gynecologic Surgery, Toulouse University Hospital, Toulouse, France

**Keywords:** Education, Surgery, Video-based learning, Surgical residency program in France, Surgical skills, Curriculum design, Medical student education, Preparedness surgical residency

## Abstract

**Introduction:**

The transition from medical student to surgical resident is not a simple one. The aim of this study was to report the experience of a university hospital in the organization of the induction course for future surgical residents and the contribution of a video support in the learning of the suture.

**Material and method:**

We were able to study two consecutive years of students (October 2020 and 2021). Concerning the practical and technical workshops (learning suture) we carried out a comparative study between two groups of students. A group that had video support for learning suture (video group) and a group without video (control group). The evaluation of the suture was performed in a blinded manner by two supervising surgeons. The other practical workshop was drain fixation; the students did not have a video for this workshop. A comparative study was also performed for the drain fixation workshop between the two groups (video group and control group). A program of theoretical courses was also set up. This program is established according to the different future functions of the residents by integrating medico-legal notions and teamwork. Satisfaction questionnaires were given to the students and the answers were given two months after taking up their duties in the hospital (6 questions with Likert scale and 4 free questions).

**Results:**

The cohort consisted of 58 students (29 each in 2020 and 29 in 2021). Comparative analyses of the evaluation of the suture workshops showed better performance in the video group compared with the group without video. The comparison of these two groups did not show significant differences in the drain fixation workshop. The theoretical teaching was broken down according to the students' future tasks and each speaker was a specialist in his or her field of expertise. The results of the questionnaires showed a desire on the part of the students to increase the time spent on practical workshops and theoretical forensic teaching.

**Conclusion:**

We were able to show through these two years of a program that we were able to offer a surgical resident preparation course. In addition, we have highlighted the contribution of a video support in the learning curve of the suture.

**Supplementary Information:**

The online version contains supplementary material available at 10.1186/s12909-022-03745-5.

## Introduction

**Fig. 1:**
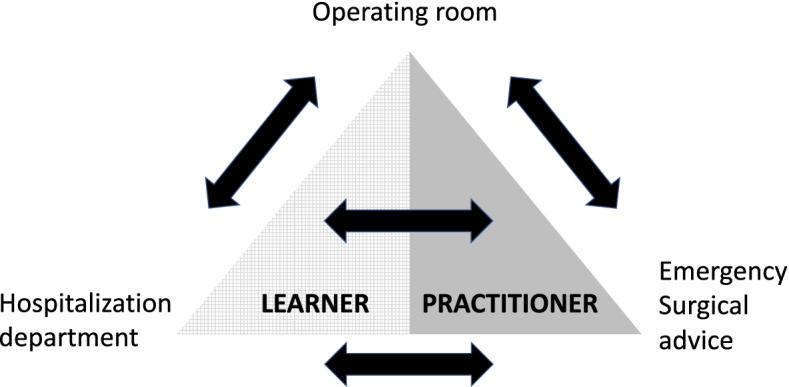
Schematic representation of the roles of surgical residents

The transition from graduate medical school to residency is often considered the most difficult transition by students and supervisors [1]. The goals of residency are to adequately perform their new clinical role, integrate into a healthcare team, and integrate their new responsibilities from day one [1]. In specialties that include a technical platform and skills such as surgical specialties, additional adaptation is required. However, despite the identification of these difficulties, the data show that students and future residents feel "underprepared" [1–3]. This phenomenon is well described in North America, particularly in Canada where it is called the "July phenomenon", and is even accompanied by a significant decrease in the efficiency of care in certain departments [4]. The purpose of such an induction program is to bridge the gap between the graduate level [5], where students are learners, and the resident level, which is a dual status of practitioner and learner (Fig. 1). The reform of the third cycle of medical studies in France now implies early choice and orientation in the specialist model requiring rapid adaptation to the student’s future surgical specialization [6]. The goal of the boot camp for future surgical residents is to introduce and prepare for technical aspects and notions related to the role and objectives of the basic phase of surgical residency. The effect of COVID-19 on medical education has led to the replacement of in-person learning with virtual experiences [7]. Thus, health restrictions and constraints have led us to reorganize to minimize the impact on students of the decrease in face-to-face teaching [8]. The aim of this study is to present the results of the seminar program at the Toulouse University Hospital. Within this program we have included the evaluation of the contribution of video support in the learning of suturing through a comparative study.

## Material and methods

### Location and context of the boot camp

The boot camp for future surgical residents in Toulouse (so called “SAICT”) takes place the week before the beginning of the first internship in a surgical department and must be attended after a specialty is chosen during the third week of September (September 18, 2020; September 17, 2021). The start of surgical residency in France takes place on November 1st and each internship lasts six months (e.g., from November to April or from May to October). For organizational and practical reasons, the workshops and classes are spread out over two days. The SAICT program is coordinated by three surgeons (two professors and one associate professor). Teaching is multi-disciplinary and includes the intervention of one or more members of the following teams: digestive surgery, pediatric surgery, vascular surgery, gynecologic surgery, the Infection Prevention and Control (IPC) team, the department of anesthesiology, the emergency department and a lawyer. The SAICT has access to the premises and infrastructures of the Toulouse Institute of Health Simulation (ITSIMS) on the Toulouse Purpan University Hospital site. The study population included all students who chose a surgical specialty available in the Toulouse University Hospital (Table 1).

**Table 1:** Student cohort

	Video group *n* = 30	Control group (without video) *n* = 28	p
Suture experience	*n* = 30 (100%)	*n* = 27 (97%)	ns
Suture and knot simulation training course	*n* = 18 (60%)	*n* = 18 (65%)	ns
Gender—male n (%)	*n* = 12 (40%)	*n* = 12 (42%)	ns
Chosen specialty	ENT and maxillofacial surgery *n* = 7	ENT and maxillofacial surgery *n* = 8	ns
	Gynecology *n* = 6	Gynecology *n* = 6	
	Digestive surgery *n* = 2	Digestive surgery *n* = 2	
	Ophthalmology *n* = 6	Ophthalmology *n* = 4	
	Cardiovascular and thoracic surgery *n* = 2	Cardiovascular and thoracic surgery *n* = 2	
	Orthopedic surgery *n* = 5	Orthopedic surgery *n* = 4	
	Urology *n* = 2	Urology *n* = 2	

*Abbreviation*: *ENT* Ear nose and throat, *ns* not significant

### Skills station

The skills stations include the first actions performed by the basic phase surgical intern. The goal of the practical workshops is to reproduce the different sequences and steps of patient management in the operating room (Table 2). These workshops are organized as follows:

- An IPC workshop focused on the prevention of surgical site infection supervised and taught by an IPC practitioner, which includes all the steps and rules of asepsis from the OR changing room to setting up sterile surgical drapes: surgical hand scrubbing, rules for setting up surgical drapes, rules for moving around the surgical theatre, skin preparation rules.
- A suture skill station supervised by the teaching surgical team. This step was comparative between two groups of students: a group with video support (Group A) one week before and another group without video support (Group B). A pre-test on the student's knowledge on theory is completed at the beginning of the seminar (Supplemental data 1). This workshop includes standardized evaluation of a suture using the Objective Structured Assessment of Technical Skills (OSATS) [9, 10] scale (Supplemental data 2). We incorporated video learning assessment using a comparative study with blinded evaluation. We created a didactic video on simple suturing and showed it to half of the cohort 3 days before the workshop. The other half did not view the video. Only one member of the teaching staff was informed of the student allocation. This team member did not participate in the theoretical and practical teaching activities. His role was to supervise the boot camp, he was not present during the suture workshops. Evaluation was performed by two members of the surgical teaching team in a blinded fashion. In case of disagreement on the evaluation, a third evaluator who was also blinded was called on. Students were randomly distributed between the two groups according to the choice of future specialty so that the two groups would be comparable for this variable.
- A drain-fixing workshop with demonstration and theory teaching was included in the seminar course. There was no video teaching for this workshop. An evaluation was also performed for this procedure with two blinded evaluators, using the Muresan scale [11, 12] (Supplemental data 3). This evaluation was also comparative between the two groups (with video and without video). No specific video was given for this skill station. The groups previously described for the suture workshop were used for the comparative study.

**Table 2:** List of SAICT boot camp skills station and lecture

		Operating room	Surgical hospitalization department	Emergency surgical advice	Lecture supervisor, teacher
Hygiene and infection	Lecture series	- Bacteriology - Guidelines for sterilization, asepsis and good practice - iatrogenic infections			Infectiology unit team member
	Skills station	- Dry and wet scrubbing - Gown and glove - Moving around the table - Prep and drape (plastic dummy)	- Dry and wet scrubbing	- Dry and wet scrubbing	
Anesthesia management	Lecture series	- Anesthetic care in the operating room - Communication between the surgeon and the anesthesiologist	- Team-based pain management - Communication between the surgeon and the anesthesiologist	- Emergency management as a team - Communication between the surgeon and the anesthesiologist	Anesthesiologist team member
	Skills station				
Handing over instruments and suture	Lecture series	- Basic knot tying and suture* - Deep knot tying and suture* - Different knot types - Instrument handling - Instrument name - Clamp-cut-tie-cut techniques			- Surgical coordination team and teaching surgeon - Member of the operating room nursing team
	Skills station	- Deep, superficial suture and knot on artificial skin			- Surgical coordination team and teaching surgeon
Drain fixation device	Lecture series	- Principles of surgical drain fixation - Principles of surgical drainage and main indications	- Drain management		- Surgical coordination team and teaching surgeon
	Skills station	- Fixation of a tubular drain (redon type) on an artificial support			- Surgical coordination team and teaching surgeon
Medical law and good practice	Lecture series	- Medical law and responsibility - Forensics rules for writing a hospitalization report and surgical report - Informed consent			- Lawyer member of the Toulouse Bar Association
	Skills station				
Team work and communication	Lecture series	- Presentation of the composition of an operating room team: roles and relationships - Duality of being a learner and practitioner	- Postoperative care with patient and family - Effective handover during transition in care	- Recognizing and initiating early management for critically ill surgical patient	- Surgical coordination team - Member of the operating room nursing team
	Skills station	Helium stick			- Surgical coordination team

### Theory courses

The theoretical part is organized into presentations of 1 h to 1 h 30 min for each session. The lessons are articulated according to the pedagogical objectives set for the SAICT. As for the skills station, each lesson is focused on taking up future functions and the tripodal operating room service of hospitalization and advice to the emergency services. The list of courses and the pedagogical objectives inherent to each are detailed in Table 2.

### Feedback from participants

At the end of the SAICT, feedback was collected from the learners. First, through a discussion with the supervisors and then in a standardized way via a questionnaire and a Likert scale which were sent to each student at the beginning of January, two months after the start of the course. The aim was to evaluate the SAICT and its efficiency in the context of new residents taking up their functions after one month of training.

### Statistical analysis

The chi-2 test, Mann–Whitney test, and Fisher’s exact test were used to analyze the demographics of the cohort. The Mann–Whitney test was used to compare unpaired nonparametric data for analysis of initial and final test results for the drain fixation and suture workshops. Data were analyzed with standard statistical tools using GraphPad-Prism 9.1.2 software (GraphPad Software Inc. San Diego, CA, USA).

## Results

### Population

The cohort included 58 students, 28 for the class of 2020 and 30 for the class of 2021 (details in the Table 1). There was no significant difference between the two groups, particularly in terms of previous experience and/or suture training.

### SAICT's program

The program for the different and practical and theory courses is detailed in Table 2.

### Results of the practical suture and drain fixation workshop evaluation

**Fig. 2:**
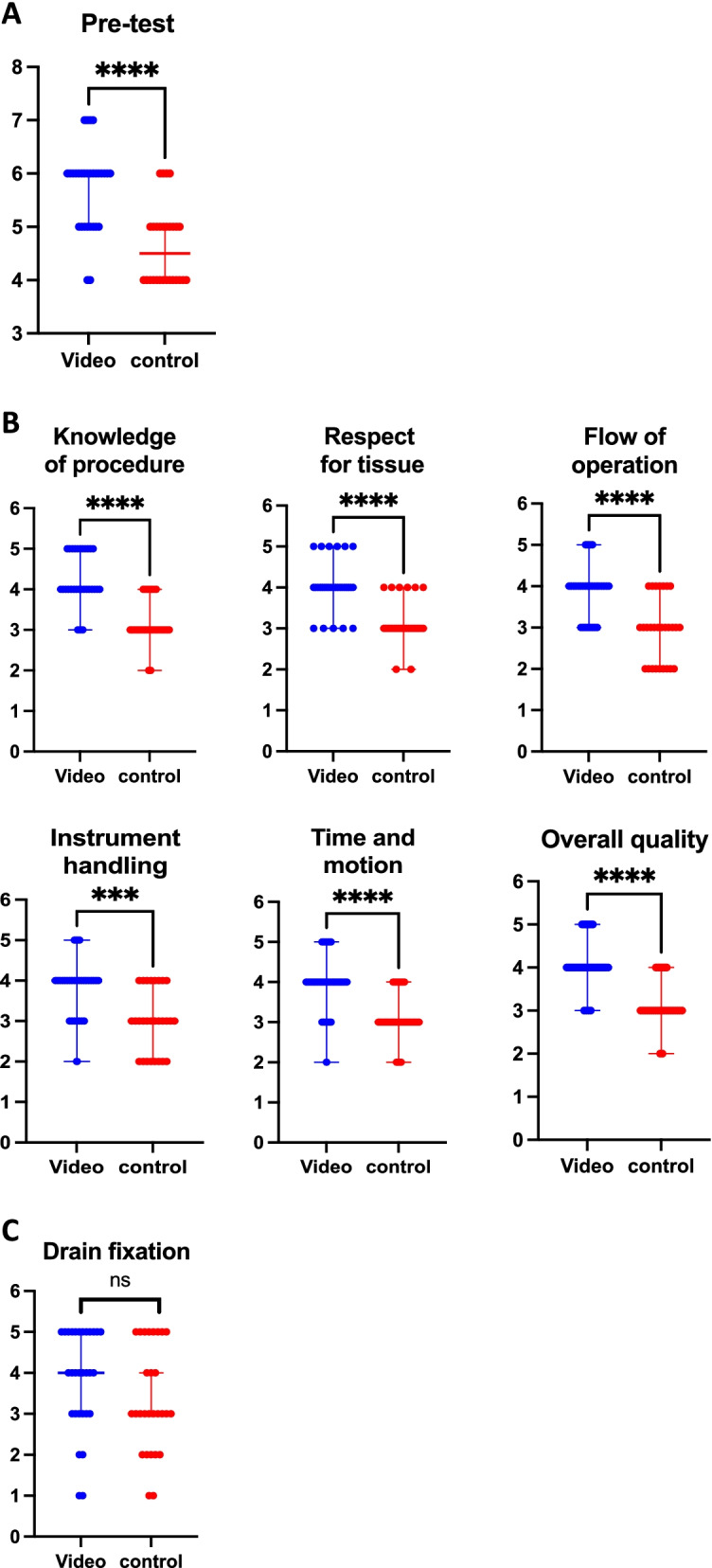
Results of the evaluation of the practical workshops; **A** results of the pre-test; **B** results of suture skills station (OSATS scale); **C** results of the drain fixation workshop (MURESAN scale)

- The pretest showed a significant difference in knowledge of procedural theory between the video and non-video groups. Knowledge of the procedure was significantly better in the video group. This result is explained by the fact that the students watched the video explaining the steps of the suture (Fig. 2A).
- The suture was evaluated on six items; each one was graded from 1 to 5. The evaluation form is detailed in supplemental data 2. The quality of the suture and of the actions was significantly better for the students in the "video" group (Fig. 2B).
- The drain fixation was evaluated on a 5-item scale of actions to take at each step (supplemental data 3). There was no significant difference between the video group and the group without a video. This evaluation was also performed blindly by a surgical examiner (Fig. 2C).

### Feedback and satisfaction questionnaire

**Fig. 3:**
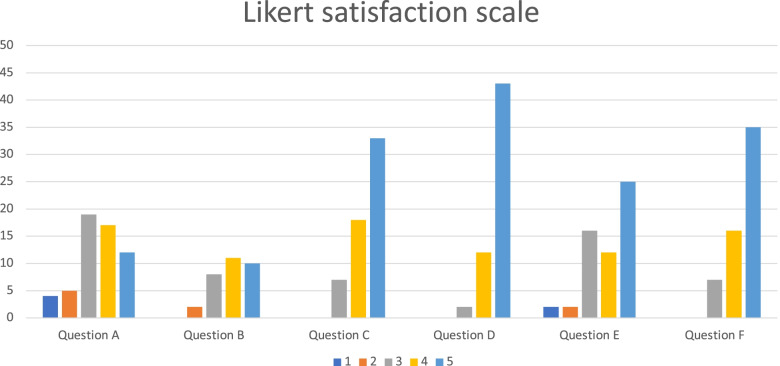
Results of the feedback questionnaire (Likert scale)

A time for discussion was included in the SAICT. Additionally, all students filled out a questionnaire in the form of a Likert scale (Table 3). The results of this questionnaire are shown in Fig. 3 (regarding question B—only students in the video group responded). In addition, the students had four additional questions (not included in the Likert scale questionnaire):

1. Which hands-on workshop would you like to increase in length?
2. Which theory workshop would you like to increase in duration?
3. Which practical workshop would you like to add?
4. Which theory workshop would you like to add?

**Table 3:** feedback questionnaire (Likert scale) regarding question B—only students in the video group responded

**Question A—Before the skills station did you identify your educational goal(s)?**				
1- Not at all	2	3	4	5- Very precisely
**Question B—Was the video useful for the suture workshop?**				
1- Not useful at all	2	3	4	5- Very useful
**Question C—Were the adjustments made by the supervisors on the use of the instruments useful at the beginning of your practice?**				
1- Not useful at all	2	3	4	5- Very useful
**Question D—How much did the hands-on suture and drain fixation skills station help you in your early practice?**				
1- Not useful at all	2	3	4	5- Very useful
**Question E—Did the presentations during the boot-camp answer your questions about the beginning of your practice?**				
1- Not at all	2	3	4	5- Very instructive
**Question F—Overall, what is your impression of the boot-camp?**				
1- Not useful at all	2	3	4	5- Very useful

Regarding the four open-ended questions, the majority of requests were:

1. 0.46 (79%) students expressed their desire to increase the duration of the practical suture workshops.
2. 0.26 (44%) students wished to increase the duration of the lessons on suture theory and 24 (41%) those on medical law.
3. 0.48 (82%) students wanted more theory lessons on the names of instruments.
4. Concerning the addition of a practical workshop, the answers were disparate and most often correlated with the orientation chosen by the students (22 students wanted a workshop on the introduction to laparoscopy, 4 on thoracic drain placement, 8 on the preparation of a plaster cast and the principles of osteosynthesis, and the remaining 22 interns from brain specialties gave disparate answers).

## Discussion

Through our results and our two years of training we showed the optimization of training time as well as the adaptation to the sanitary constraints. In fact, the efficiency of the video support for sutures indicates that time for theory lessons on this subject can be reduced. Through this optimization, the time and number of skills stations and theory courses can be increased. The objective is always to prepare residents in the best possible way to take up their future functions [13]. The reform of the third cycle of medical studies in France now divides internship into three distinct parts: the foundation phase, the enhancement phase and the consolidation phase, each of which is spread out over two years, for a total of six years. During the foundation phase, resident must complete their practice in a tertiary center of their future specialty, which implies early specialization. The SAICT allows the introduction of more general notions of surgery such as the use of instruments or notions of medical law required for daily practice (e.g. writing prescriptions, medical observations, the notion of professional secrecy).

One of the first areas requiring change for future surgical residents is the operative and execution side of daily practice. This aspect is also a source of apprehension. Therefore, progressive learning in a safe environment seemed appropriate [14]. The construction of the motor pattern is an essential element in the development of future technical skills [12]. Our cohort suggests that watching a video on how to suture contributes significantly to learning. In fact, we found significant differences in all items of the OSATS [10]. One of the difficulties encountered during this SAICT project was related to the health constraints of the COVID 19 pandemic [15]. This constraint allowed us to establish and refine our method of learning how to suture through a video. This method proved to be effective and will allow us to save time and implement our theory teaching program through other lessons. The essential elements of this practical lesson are a debriefing stage by the supervisors [16], out-of-hospital boot camp and, above all, a preparation stage appears to be essential [1]. Debriefing was considered important here because this kind of process has already been proven to work in emergency situations and technical situation [17, 18]. Introducing debriefing concepts early in surgical training can help to establish a habit and adapt it for increasingly technical procedures as the training progresses. The post-study Likert survey suggests that users appreciated the tool, feeling that it improved their early practice.

Concerning the follow-up and evolution of resident learning, it is integrated in the basic phase of the practical teaching in the form of a seminar every two months during the first year. These seminars allow the residents to give feedback on the technical procedures and to express any difficulties they may have in performing them. Our model and the changes in French residency teaching allow us to have a follow-up over the year [6]. This has the advantage of having feedback from all the students, especially on the Likert scales. On the learners' side, this allows an evolutive follow-up and adaptations of the SAICT each year according to the needs expressed by the residents during the SAICT and throughout the first year of practice. We can thereby follow residents in digestive, pediatric, gynecological, vascular and urological surgery by progressively integrating learning workshops, laparoscopic surgery and practical workshops in digestive and vascular anastomosis [19].

For the 2021 session, we also conducted a survey among the heads of the different surgical specialties in order to find out their expectations regarding the new interns taking up their duties in their departments, as well as their expectations, if any, regarding this seminar. We observed that the students were eager to increase the duration, the number and the type of practical sessions. To this end, the SAICT 2022 will be enhanced with an additional "more focused" day according to the various specialties. Future residents will be assigned to theory lessons and practical workshops according to their specialties. These days will be supervised by teachers of the respective specialties.

## Conclusion

The SAICT contains a wealth of new information concerning both theory and practice that future resident must acquire. In addition, we have highlighted the contribution of a video support in the learning curve of the suture. Based on the results of the SAICT, we were able to show that it is possible to organize an induction course that could cover the basic notions required by surgical residents before they take up their function. The SAICT will be implemented by consolidating the general bases common to all future residents, regardless of their specialty, as well as through targeted and specialized teaching provided by specialty teachers.

## Supplementary Information

**Additional file 1.**

## Data Availability

The data sets used and/or analysed during this study are available from the corresponding author on request.
