# Supplementary material for: Boot camp approach to surgical residency preparation: feedback from a French university hospital
Source: BMC Med Educ. 2022 Sep 15;22:678. doi: 10.1186/s12909-022-03745-5 (PMC9473737; doi:10.1186/s12909-022-03745-5)
Supplement: Supplementary file 1 — Additional file 1. [file 12909_2022_3745_MOESM1_ESM.docx]

**Supplemental data**

*Supplemental data 1: pre-test (one correct answer to each question)*

Question 1: The most penetrating needle is:

A) Triangular section

B) Round section

C) A 5/8 curvature

Question 2: Which of the following needles is it best to use to pass a stitch deep into a space:

A) Straight

B) 3/8

C) 4/8

D) 5/8

Question 3: The finest thread is:

A) 10/0

B) 2/0

C) 1

Question 4 Absorbable sutures :

A) Are dissolved or digested by tissues in the human body

B) Lose their strength after 2 days

C) Are all braided threads

Question 5: A needle holder is held:

A) Between the thumb and forefinger

B) By inserting the fingers deep into the eyelets

C) Between the thumb and ring finger

Question 6: A pair of dissecting forceps

A) Is held in the dominant hand during suture

B) Is always clawed to better grasp the tissue

C) Is held like a pen

Question 7: A good quality stitch

A) Is very tight

B) Equidistant from each edge

C) The depth must be greater on the side of the needle holder

D) Always has 5 knots

*Supplemental data 2: Objective Structured Assessment of Technical Skills*

*OSATS scale*

|  | 1 | 2 | 3 | 4 | 5 |
| --- | --- | --- | --- | --- | --- |
| Respect  for tissue | Frequently used unnecessary force on tissue or caused damage by inappropriate use of instrument |  | Careful handling of tissue but occasionally caused inadvertent damage |  | Consistently handled tissues appropriately with minimal damage |
| Time and motion | Many unnecessary moves |  | Efficient time/motion but some unnecessary moves |  | Economy of movement and maximum efficiency |
| Instrument handling | Repeatedly makes tentative or awkward moves with instruments |  | Competent use of instruments although occasionally appeared stiff or awkward |  | Fluid movement with instrument and no awkwardness |
| Flow of operation | Frequently stopped or needed to discuss next move |  | Demonstrated ability for forward planning with steady progression of operative procedure |  | Obviously planned course of operation with effortless flow from one move to the next |
| Knowledge of procedure | Deficient knowledge. Needed specific instruction at most operative steps |  | Knew all important aspects of the procedure |  | Demonstrated familiarity with all aspects of the operation |
| Overall quality | Very poor |  | Medium |  | Perfect |

*Supplemental data 3: Muresan scale Scoring system for knot quality assessment*

| Quality assessment | Available points |
| --- | --- |
| No visible gaps between stacked rows | 1 |
| Knot tight at base | 1 |
| Only edges are opposed | 1 |
| Knot hold under tension | 2 |
| Maximum points | 5 |
